# Identification of the ancestral killer immunoglobulin-like receptor gene in primates

**DOI:** 10.1186/1471-2164-7-209

**Published:** 2006-08-15

**Authors:** Jennifer G Sambrook, Arman Bashirova, Hanne Andersen, Mike Piatak, George S Vernikos, Penny Coggill, Jeff D Lifson, Mary Carrington, Stephan Beck

**Affiliations:** 1 Immunogenomics Laboratory, Wellcome Trust Sanger Institute, Genome Campus, Hinxton, Cambridge CB10 ISA, UK; 2 Laboratory of Genomic Diversity, National Cancer Institute, SAIC-Frederick, MD 21702, USA; 3 Retroviral Pathogenesis Laboratory, AIDS Vaccine Program, SAIC-Frederick, MD 21702, USA; 4 Laboratory of Genomic Diversity, Basic Research Program, SAIC-Frederick, MD 21702, USA; 5 current affiliation: Department of Infectious Diseases, School of Medicine, Johns Hopkins University, Baltimore, MD 21231, USA; 6 current affiliation: BIOQUAL Inc, Rockville, MD 20850, USA

## Abstract

**Background:**

Killer Immunoglobulin-like Receptors (KIR) are essential immuno-surveillance molecules. They are expressed on natural killer and T cells, and interact with human leukocyte antigens. KIR genes are highly polymorphic and contribute vital variability to our immune system. Numerous KIR genes, belonging to five distinct lineages, have been identified in all primates examined thus far and shown to be rapidly evolving. Since few KIR remain orthologous between species, with only one of them, *KIR2DL4*, shown to be common to human, apes and monkeys, the evolution of the KIR gene family in primates remains unclear.

**Results:**

Using comparative analyses, we have identified the ancestral KIR lineage (provisionally named *KIR3DL0*) in primates. We show *KIR3DL0 *to be highly conserved with the identification of orthologues in human (*Homo sapiens*), common chimpanzee (*Pan troglodytes*), gorilla (*Gorilla gorilla*), rhesus monkey (*Macaca mulatta*) and common marmoset (*Callithrix jacchus*). We predict *KIR3DL0 *to encode a functional molecule in all primates by demonstrating expression in human, chimpanzee and rhesus monkey. Using the rhesus monkey as a model, we further show the expression profile to be typical of KIR by quantitative measurement of *KIR3DL0 *from an enriched population of natural killer cells.

**Conclusion:**

One reason why *KIR3DL0 *may have escaped discovery for so long is that, in human, it maps in between two related leukocyte immunoglobulin-like receptor clusters outside the known KIR gene cluster on Chromosome 19. Based on genomic, cDNA, expression and phylogenetic data, we report a novel lineage of immunoglobulin receptors belonging to the KIR family, which is highly conserved throughout 50 million years of primate evolution.

## Background

The Killer Immunoglobulin-like Receptor (KIR) gene family encodes Major Histocompatibility Complex (MHC) class I specific receptors that are expressed on Natural Killer (NK) and T cells [1,2]. In humans, these are encoded within the Leukocyte Receptor Complex (LRC) [3] on Chromosome 19q13.4, which like the MHC on Chromosome 6p21.3, is a region characteristic of immune loci: highly plastic, polygenic, polymorphic, rapidly evolving, and associated with disease [4]. As a result, KIR diversity contributes vital variability to our immune system with direct implications for health and disease [5,6]. KIR working in concert with its Human Leukocyte Antigen (HLA) ligands has been shown to influence directly the resolution of viral infections such as Hepatitis C Virus [7].

Numerous KIR genes, both in their activating and inhibitory forms, have been identified in all primates examined thus far and shown to be rapidly evolving [8-11]. Inhibitory KIR have longer cytoplasmic tails in comparison to activating KIR, and typically contain two immunoreceptor tyrosine-based inhibitory motifs (ITIMs) which are responsible for repressing the immunoreactivity of NK cells. The emergence of primate activating KIRs can be accounted by two processes: the alteration in the length and sequence of the cytoplasmic tail in an ancestral long-tailed KIR to eliminate the ITIMs, accompanied by nucleotide changes in the transmembrane (TM) domain to introduce a charged residue [12]. Another distinguishing feature of KIR molecules is the number of extracellular immunoglobulin (Ig) domains, numbered D0, D1 and D2. Although *KIR2DL4 *is conserved in all primates studied to date [11], it is unlikely to represent the ancestral KIR gene. Structurally, *KIR2DL4 *has a D0+D2 organisation and has arisen by exon loss from a three Ig-containing progenitor. The ITIMs present in the cytoplasmic tail are also not conserved between all primates: monkeys have two, apes have only one, and in some cases, the motif has diverged from the consensus (gorilla) [13]. *KIR2DL4 *has a charged amino acid in the TM region, and in this respect, could act as an activating KIR [8]. It binds the non-polymorphic HLA-G molecule, and this may explain why this gene has remained relatively unchanged since the last common ancestor. Here we report a novel lineage (provisionally named *KIR3DL0*) and show it to be divergent to the previously identified lineages and conserved throughout 50 million years of primate evolution. Characteristics that would be expected to be present in the common ancestral primate KIR, such as three Ig domains, a long cytoplasmic tail, and two ITIMs providing an inhibitory function, are all present in the *KIR3DL0 *lineage. For the purpose of this report, we define 'ancestral' as the phylogenetically most diverged gene. In support of this discovery, we present genomic, cDNA, expression and phylogenetic data.

## Results and discussion

### Identification of the KIR3DL0 locus

**Figure 1:**
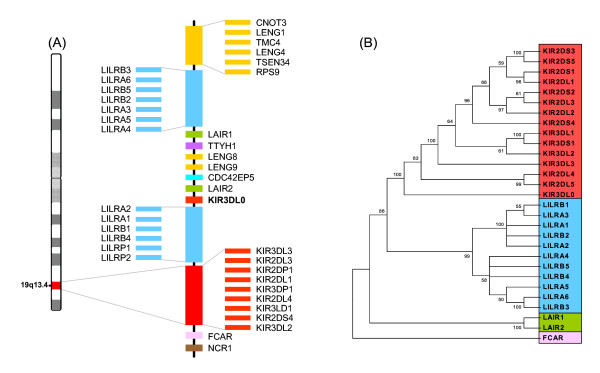
**(A) Location of *KIR3DL0 *in the human LRC on chromosome 19q13.4**. The LRC comprises a number of diverse genes and gene clusters including leukocyte Ig-like receptors (LILRs), killer Ig-like receptors (KIRs), LRC-encoded novel genes (LENGs) and leukocyte-associated Ig-like receptors (LAIRs). Human KIR haplotypes differ in gene content; only the prototypical haplotype A is shown. (**B**) Phylogenetic analysis using complete protein sequences of *KIR3DL0 *and other Ig-domain containing receptors in the human LRC.

Although still located within the LRC [3,14], the human *KIR3DL0 *locus maps approximately 180 kb centromeric to the known KIR cluster (Figure 1A). This location in between the two related leukocyte immunoglobulin-like receptor (LILR) clusters may explain why this gene has escaped discovery for so long. Despite its location outside of the KIR cluster, *KIR3DL0 *is clearly more related phylogenetically to KIR genes than to any other Ig-containing gene within the LRC (Figure 1B).

**Figure 2:**
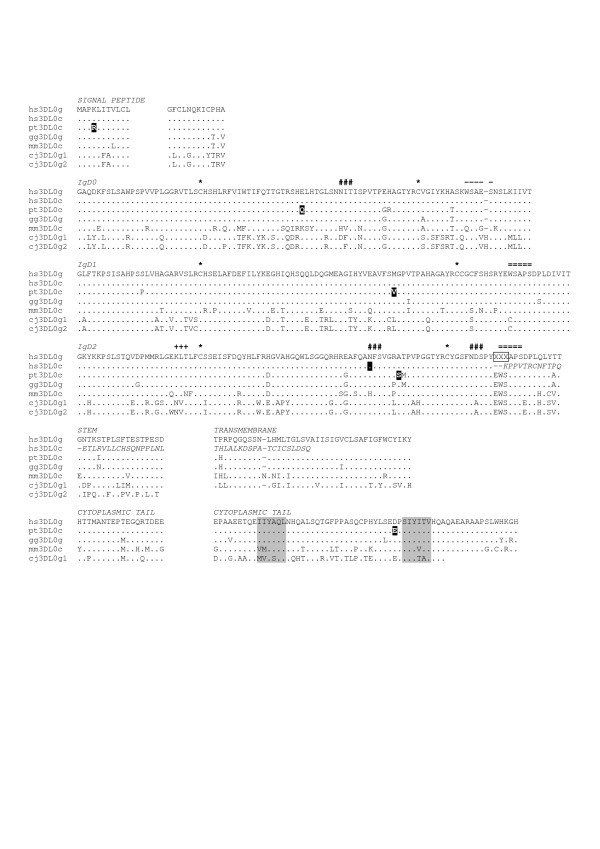
***KIR3DL0 *protein sequences in primates**. The multiple sequence alignment includes human (hs), common chimpanzee (pt), gorilla (gg), rhesus monkey (mm) and common marmoset (cj). The suffixes 'c' and 'g' denote cDNA-derived or predicted from genomic sequence, respectively. Sequences are split according to their exonic boundaries. The frameshift causing deletion of seven bases in IgD2 of the human gene has been manually corrected by insertion of three Xs (boxed) to bring the sequence back into frame. The human cDNA sequence is shown in italics after the frameshift as it is not part of the multiple sequence alignment. Dots (.) indicate identity with human *KIR3DL0g *and dashes (-) indicate insertions/deletions of amino acids. Inversely labeled amino acids designate polymorphic positions where one allele is identical to *hsKIR3DL0g *and another as indicated. Inhibitory motifs (ITIM) are highlighted in grey. Asterisks (*) indicate cysteine residues that are likely to form disulfide bridges. N-linked glycosylation signals (NXS/T) are shown by hashes (#) when conserved between the majority of primate sequences and plus signs (+) when present in only marmoset. Putative beta-bulges containing the WSXS/PS or L/VSAPS motifs are delineated by double bars (=) while single bars (-) mark partially conserved motifs. Two adjacent copies of *KIR3DL0 *are present in marmoset with one of them (*cjKIR3DL0g2*) missing the last three exons.

All KIR family members share a comparable gene structure; discrete exons encode the signal peptide, either two or three extracellular immunoglobulin domains, a stem, transmembrane region, and a cytoplasmic tail. As we show below, the genomic sequence for *KIR3DL0 *is characteristic of KIR and highly conserved in all primates (Figure 2), but, in human, a single deletion of seven nucleotides at the end of the third immunoglobulin domain (IgD2) in exon 5 leads to a frameshift, preventing it from being expressed on the cell surface. Because of the deletion, it uses alternative reading frames for the end of exon 5 and exons 6/7, resulting in a potentially secreted form of *KIR3DL0 *with no TM and cytoplasmic domains. The only possibility of the *KIR3DL0 *receptor remaining membrane-bound would be an association with the adaptor molecule DAP12 mediated by a charged amino acid in the TM. DAP12 is able to stimulate cytotoxic activity as it contains an immunoreceptor tyrosine-based activation motif and is the mode in which activating KIRs have an effect. Although the frameshift has resulted in the presence of a charged amino acid (K) in the terminal exon (exon 7), the deduced amino acid sequence does not correspond to a TM or core hydrophobic region, which makes the association with DAP12 less likely in this case. Human *KIR3DL0 *is based on a full-length cDNA isolated from a human NK cell line (NK-92), and is further supported by a partial cDNA sequence [GenBank:BC033195] from the mammalian gene collection (MGC), isolated from a different human NK cell line (NIH_MGC_106). In order to check for polymorphism, 86 unrelated healthy individuals were sequenced across the deleted region and the same frameshift causing deletion was present in all individuals.

The relatedness of *KIR3DL0 *to the KIR gene family is further confirmed by gene structure analyses. Using FINEX, a method that identifies gene families based on the conservation of gene structure (intron/exon phases) rather than sequence similarity [15], the closest relative of *KIR3DL0 *is the prototypical human KIR gene *KIR3DL1 *with a significant z-score of +10.88 (data not shown). Furthermore, using VISTA [16], a global genomic comparison tool, significant conservation in both coding and non-coding sequences is present between all primate *KIR3DL0 *sequences and *KIR3DL1 *[see Additional file 1].

### Conservation of KIR3DL0 in primates

We show *KIR3DL0 *to be highly conserved in primates with the identification of orthologues in the common chimpanzee (*Pan troglodytes*), gorilla (*Gorilla gorilla*), rhesus monkey (*Macaca mulatta*) and common marmoset (*Callithrix jacchus*). The two copies in marmoset are arranged in a head-to-head orientation and are likely to be the result of a species-specific duplication event. These non-human primates diverged from humans approximately 5, 7, 25 and 50 million years ago, respectively. In all cases, *KIR3DL0 *maps to the syntenic region between *LAIR2 *and *LILRA2*. Details of the genomic LRC organization in these primates will be described elsewhere.

The primate *KIR3DL0 *sequences share the same exon-intron configuration and are predicted to encode functional proteins (Figure 2). In addition to the human cDNA, full length cDNA sequences were isolated from chimpanzee and rhesus monkey. Variations between the genomic and cDNA sequences may indicate *KIR3DL0 *to be polymorphic. Although only two sequences could be compared per species, we identified five non-synonymous substitutions in chimpanzee and one synonymous substitution in human. The conservation of two immunoreceptor tyrosine-based inhibitory motifs, present in the cytoplasmic tail of the predicted *KIR3DL0 *protein sequences from all species studied, indicates an inhibitory function for these receptors. These motifs are present in only one copy in marmoset, which has the complete nine-exon gene structure. The short form is missing the last three exons encoding the TM and cytoplasmic domains, resulting in a potentially secreted form, which is similar, but not identical, to the *KIR3DL0 *cDNA in human (Figure 2). While three N-linked glycosylation sites (NXS/T) are conserved in the majority of the sequences analyzed, one additional site is present in the IgD2 of the two *KIR3DL0 *copies in marmoset. For the short marmoset form in particular, the extra glycosylation site increases the potential level of glycosylation. High levels of glycosylation have been shown to be essential for secretion of some immunoglobulins [17].

### Phylogenetic analysis

**Figure 3:**
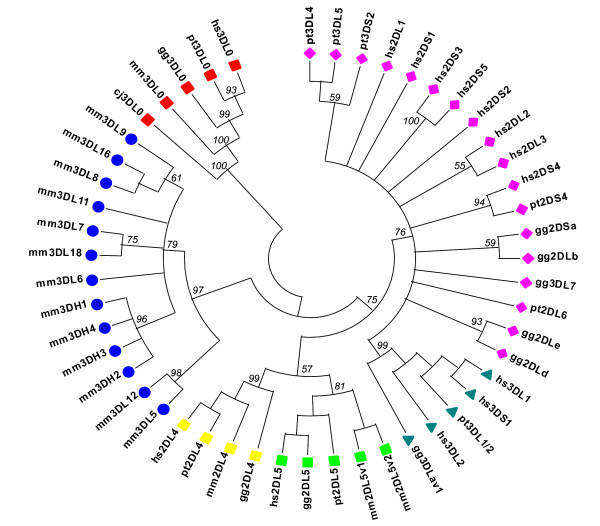
**Phylogenetic analysis of KIR genes in primates**. Sequences for human (hs), common chimpanzee (pt), gorilla (gg), rhesus monkey (mm) and common marmoset (cj) have been grouped according to their lineages: lineage 1A (yellow squares), lineage 1B (green squares), lineage II (green triangles), lineage III (pink diamonds), lineage IV (blue circles) and the newly proposed lineage 0 encompassing the novel *KIR3DL0 *genes is marked by red squares. Lineage V genes were excluded from the analysis owing to their variable phylogenetic clustering and varied exon organization [8]. Phylogenetic analysis was carried out on protein sequences that were edited manually to maximise the alignment between corresponding Ig domains, as well as the stem, TM and cytoplasmic tail.

The KIR genes identified to date have been divided into five distinct lineages [8,9,11,13]: lineage I comprises genes with specific domain configurations (IgD0 and IgD2 domains), lineage II is defined by ligand specificity (*HLA-A *and *HLA-B*), lineage III contains a mixture of KIR genes with two or three Ig domains, lineage IV genes are only found in rhesus monkeys, and lineage V encompasses KIR genes lacking the stem. Having established that *KIR3DL0 *is conserved across species at the gene and protein levels (e.g. 33–41% similarity to human *KIR3DL1 *[see Additional file 2], we used phylogenetic analysis to determine whether or not the new *KIR3DL0 *genes also represent a novel KIR lineage. Using the deduced full-length protein sequences of known primate KIR genes, the tree shown in Figure 3 was constructed. The novel *KIR3DL0 *sequences all cluster separately and with high bootstrap confidence at the base of the tree, demonstrating that *KIR3DL0 *represents not only a novel but also the ancestral lineage of primate KIR genes. In order to confirm this finding and to assess whether, because of its location, *KIR3DL0 *arose by non-homologous recombination between KIR and LILR sequences, separate trees were also constructed for each of the *KIR3DL0 *domains and the corresponding domains of KIR and LILR. Under the conditions used here (see Materials and Methods for details), all *KIR3DL0 *domains clustered at the base of the KIR clade, separated from the LILR domains [see Additional file 3]. The IgD1 domain of *KIR3DL0 *is more closely related to the IgD0 domains of the KIR gene family (including the IgD0 of *KIR3DL0*) in comparison to all other domains used in the analysis.

### Expression profiles

**Figure 4:**
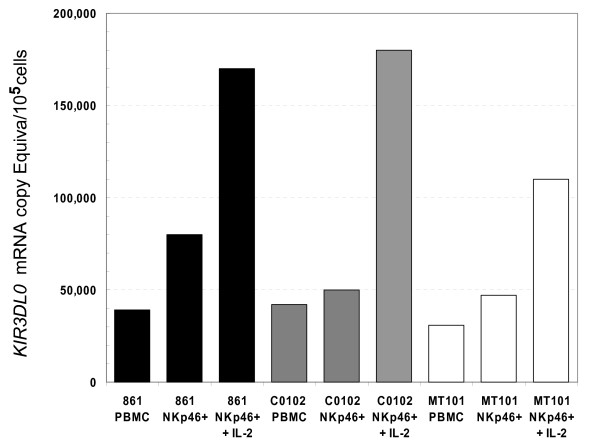
**Expression analysis of *KIR3DL0 *in rhesus monkey**. The figure shows normalized *KIR3DL0 *mRNA expression in PBMC, NKp46+ cells, and NKp46+ cells cultured for one week with 100 U/mL recombinant human IL-2 for cells from three different rhesus monkeys, 861 (black), C0102 (gray), and MT101 (white).

We confirmed expression of *KIR3DL0 *by cDNA cloning in human, chimpanzee and rhesus monkey. The human cDNA was cloned from an NK cell line (NK-92), and preliminary analysis shows expression in five other NK cell lines but not in Jurkat cells (T cells) (data not shown). To evaluate the pattern of expression in more detail, we chose the rhesus monkey. Peripheral blood mononuclear cells (PBMC) were obtained and NKp46+ cells, previously shown to be enriched for NK cytolytic activity [18], were isolated by immunoaffinity methods. Firstly, using RT-PCR, a 1.4 kb cDNA was identified that encodes a protein with 458 amino acids, and verifies the *in-silico *structure of primate *KIR3DL0*. A second fragment was also observed and represents an alternative splice form missing exon 5; this variant has only two Ig domains. Secondly, levels of *KIR3DL0 *mRNA in these cell populations were quantified by a real time RT-PCR assay. In view of data indicating that *IL-2 *can increase expression of KIRs, rhesus NKp46+ cells were also cultured in the presence of recombinant human *IL-2 *for one week, and tested for *KIR3DL0 *mRNA expression at the end of this period. As shown in Figure 4, levels of *KIR3DL0 *message were modestly enriched in NKp46+ cells compared to unfractionated PBMC for the three rhesus monkeys tested, although the relatively low number of copies per NKp46+ cell suggests that the receptor may be expressed by only a subset of NKp46+ cells. Culture in the presence of *IL-2 *resulted in substantial upregulation of expression of *KIR3DL0 *in cells from all three animals.

## Conclusion

Based on comparative sequence analysis, we have identified a new lineage of immunoglobulin receptors and show them to be part of the KIR receptor family, supported by four independent lines of evidence. (I) Sequence similarity: Using the BLAST algorithm, the similarity on both the DNA and protein level is highest to KIR. (II) Gene structure similarity: The nine exon gene structure and the phases of the splice site boundaries are identical or more similar to KIR than to any other gene family. (III) Phylogenetics: Full length and domain-by-domain phylogenetic analysis cluster the new sequences with high confidence at the base of the KIR clade. (IV) Expression: cDNA cloning and quantitative RT-PCR analysis demonstrate an expression profile typical of KIR. While the data presented here suggest *3DL0 *to be the ancestral KIR gene in primates, we cannot rule out the possibility of selection to have caused the observed divergence. The situation in non-primates remains unclear. Based on our own and previously reported phylogenetic evidence [12], the primordial non-primate KIR gene would be predicted to be a three-Ig inhibitory receptor, most similar to *KIR3DL0*. Yet, we were unable to find any significant matches to *KIR3DL0 *in non-primates, except to previously reported sequences [19-23] that have higher similarity to more recent KIRs. Recently, a cluster of CHIR genes has been identified in chicken, but these resemble the organisation of two-domain KIRs [24]. A possible explanation for these observations is that *KIR3DL0 *has been lost from non-primate genomes. Polyphyletic loss of multiple human genes from non-primate vertebrate genomes has been observed before and has led to the suggestion that such events are more frequent than previously thought [25]. Both rapid and convergent evolution have probably contributed to or even driven this process. In rodents, for instance, multiple *Ly49 *genes, which encode C-type lectins, carry out the KIR analogous function. The two murine *Kirl1 *and *Kirl2 *genes [21] lack the characteristic activating/inhibitory motifs of KIRs and map to the X chromosome outside the otherwise conserved LRC. Likewise, the single KIR-like sequence in rat (*Kir3dl1*) [19] lacks the stem region and has an elongated TM region. The sequence has, however, the same overall (3DL) structure as *KIR3DL0 *and maps to the syntenic region in the rat LRC and, in this respect, represents the closest non-primate relative to *KIR3DL0*.

## Methods

### Mapping and sequencing

BAC contigs covering the KIR region were generated and sequenced as described previously [11]. *KIR3DL0 *genomic sequences were obtained as follows: for human, using ENSEMBL [26] assembly NCBI-35; for chimpanzee, using ENSEMBL assembly CHIMP-1; for gorilla, by BAC sequencing [EMBL:CR759947 and EMBL:CR759950]; and for marmoset, by BAC sequencing [EMBL:CR925829]. The single sequence gap that covers the transmembrane region in the CHIMP-1 assembly was amplified and sequenced using forward (5'-caccaacattctttggagcaagtt-3') and reverse (5'-aggctgaggtggaagaatggc-3') primers. The frameshift region in the human *KIR3DL0 *gene was sequenced in 86 healthy individuals (81 Caucasian, 3 African American, 1 Asian, 1 unknown ethnicity) using forward (5'-ttccaggccaacttttctgtgg-3'), and reverse (5'-tctgtgatccagtgggcacca-3') primers. *KIR3DL0 *cDNA sequences were obtained by reverse transcription of total RNA using Superscript (Invitrogen). Total RNA was isolated using Trizol (Invitrogen). The macaque and chimpanzee *KIR3DL0 *cDNAs [GenBank:DQ224422 and GenBank:DQ157756] were cloned by two rounds of PCR from peripheral blood lymphocyte cDNA. The first round of PCR was performed with primers KIR3DL0-F2, 5'-ctgtgtcctgcccaatagaag-3', and KIR3DL0-R2, 5'-cctcctaggaatagatccagg-3'. For the second round, we used primers KIR3DL0-F2 and KIR3DL0-R1, 5'-aggaatagatccaggtccttg-3'. The human *KIR3DL0 *cDNA [GenBank:DQ224421] was cloned by one round of PCR using the KIR3DL0-F2 and KIR3DL0-R1 primers.

### Sequence analysis

Genomic sequences were analyzed using ARTEMIS [27], ACT [28], and BLAST [29]. Multiple sequence alignments were carried out in ClustalX [30], and edited manually to maximize the alignment. Trees were constructed using the Neighbour Joining method [31] in MEGA version 2.1 [32], using human *KIR3DL0 *derived from genomic sequence, with the frameshift in IgD2 manually corrected to bring the sequence back into frame. Trees were rooted at midpoint, using a complete deletion (Figure 1B) or pairwise deletion (Figure 3), the Poisson correction option, and 500 bootstrap replicates. Global genomic comparisons were made using VISTA [16]. FINEX [15] was used in conjunction with its corresponding gene structure database derived from EMBL version 181 (eukaryotes only).

### Expression analysis

#### Isolation and culture of rhesus macaque PBMC and NKp46^+ ^lymphocytes

Peripheral blood mononuclear cells were isolated from rhesus macaque as described previously [18]. To isolate an enriched NK cell population, PBMC were first incubated with a PE-conjugated anti-human NKp46^+ ^mAb (clone BAB281, Beckman Coulter, Miami, FL), washed once, incubated with PE-selection beads (Miltenyi, Auburn, CA) and passed over a positive selection column for enrichment of PBMC expressing the NKp46 surface marker. Successful enrichment was verified by flow cytometric analysis using a FACSCalibur (Becton Dickinson, Immunocytometry Systems, San Jose, CA); selected cells were ≥85% NKp46^+^. To assess induction of *KIR3DL0 *expression following *in vitro *culture with IL-2, NKp46^+ ^cells were cultured in RPMI-10% FBS with 100U/ml recombinant human IL-2 (Peprotech, Rocky Hill, NJ) for 7 days. Cells were counted, transferred into 1.5 ml RNAse-free polypropylene tubes, spun down, and snap-frozen in liquid nitrogen.

#### Nucleic acid extraction and quantitative PCR/RT-PCR analysis

Cellular nucleic acids for qPCR and qRT-PCR were prepared with modifications to methods typically applied to the isolation of RNA from plasma associated simian immunodeficiency virus [33]. The disruption and suspension of cell pellets in lysis buffer was facilitated by sonication using a Branson Model S-450D sonifier equipped with a high intensity cup horn (Branson Ultrasonics, Corp., Danbury, CT). The final nucleic acid containing pellet was dissolved in 60 μl 1X Turbo DNase Buffer (Ambion, Inc., Austin, TX) and divided into two equal aliquots. To one aliquot, 2 units of Turbo DNase (Ambion, Inc., Austin TX) were added and the aliquot incubated at 37°C. After 30 minutes, 120 μl ~5.7 M GuSCN, 50 mM TrisCl, pH 7.6, 1 mM EDTA was added and the sample mixed thoroughly, followed by 150 μl isopropanol to precipitate and recover nucleic acid. This sample was dissolved in 30 μl nuclease free water supplemented to contain 1 mM DTT and 1 U/ml RNASeOUT (Invitrogen, Inc., Carlsbad, CA) and assayed by quantitative RT-PCR methods following the conditions and protocols described in Cline et al. [33] with the following modifications to accommodate the increased level of cellular RNA and to specifically amplify the target *KIR3DL0 *mRNA sequence: in the reverse transcription step, 1.5 μg random primers and 100 Units reverse transcriptase per reaction were employed and the 85°C heat-kill step was increased to 25 minutes; in the PCR step, the primers and probe used were *KIR3DLO*-FOR02, 5'-tgggagcaaatccctgaagat-3', *KIR3DLO *-REV02, 5'-agcttgggtgcgctgagat-3', and *KIR3DLO *-PR02, 5'-FAM-tcgtcacaggcttgtttaccaaaccctc-BlackHole™ Quencher 1 (Biosearch Technologies, Novato, CA). The remaining aliquot of nucleic acids was heated to 100°C for 5 min, quenched on ice, and then assayed to determine the number of cell genome equivalents represented in the original sample by quantifying copies of a gene sequence for the chemokine receptor CCR5 (MP, unpublished data).

## Abbreviations

Human Leukocyte Antigen (HLA); immunoglobulin (Ig); immunoreceptor tyrosine-based inhibitory motifs (ITIM); Killer Immunoglobulin-like Receptor (KIR); Leukocyte Immunoglobulin-like Receptor (LILR); Leukocyte Receptor Complex (LRC); Major Histocompatibility Complex (MHC); peripheral blood mononuclear cells (PBMC); transmembrane (TM)

## Authors' contributions

JGS, GSV, PC carried out the mapping and sequence analysis. JGS drafted the manuscript. AB carried out the cDNA analysis and interpretation of data. HA, MP, JDL performed the expression profiles. SB and MC participated in the design and coordination of the project, and helped to draft the manuscript. All authors have read and approved the final manuscript.

## Supplementary Material

Additional File 1 **VISTA plot of human *KIR3DL1 *and *KIR3DL0 *in primates**. The VISTA plot compares sequence conservation between human *KIR3DL1 *and *KIR3DL0 *in human, chimpanzee, gorilla and marmoset. Click here for file

Additional File 2 **Sequence similarities between human *KIR3DL1 *and *KIR3DL0 *in primates**. The table shows nucleotide and amino acid sequence similarities between human *KIR3DL1 *and *KIR3DL0 *in human, gorilla, chimpanzee, rhesus monkey and marmoset. Click here for file

Additional File 3 **Domain-by-domain analyses of human KIR and LILR genes**. Phylogenetic trees are shown for the Ig domains, signal peptide, and the combined sequence of the stem, transmembrane and cytoplasmic tail. Click here for file
