# Supplementary material for: Identification of the ancestral killer immunoglobulin-like receptor gene in primates
Source: BMC Genomics. 2006 Aug 15;7:209. doi: 10.1186/1471-2164-7-209 (PMC1559706; doi:10.1186/1471-2164-7-209)
Supplement: Additional File 1 — VISTA plot of human KIR3DL1 and KIR3DL0 in primates. The VISTA plot compares sequence conservation between human KIR3DL1 and KIR3DL0 in human, chimpanzee, gorilla and marmoset. [file 1471-2164-7-209-S1.pdf]

Additional file 1. VISTA plot of human *KIR3DL1* against *KIR3DL0* in primates.

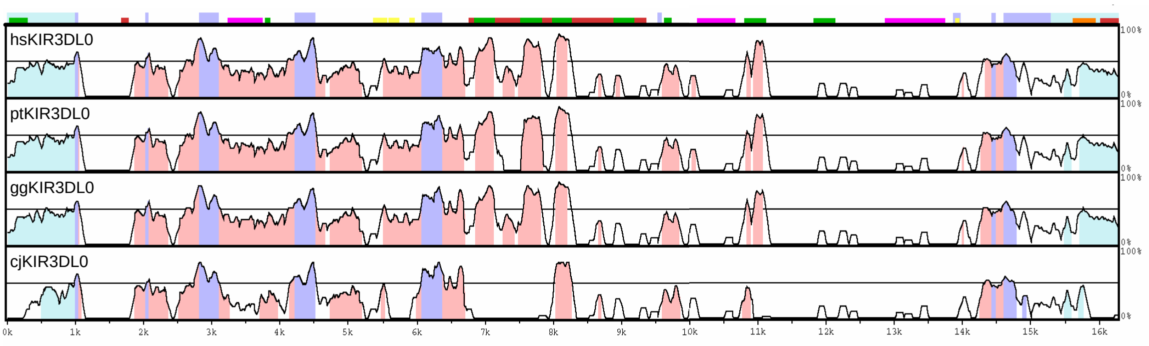

Sequences for human (hs), chimpanzee (pt), gorilla (gg) and marmoset (cj) were compared using the MLAGAN alignment program. Conserved regions with more than 30% sequence similarity over a 100 base pair window are colored; pale-pink regions are conserved non-coding sequences, purple regions are exons, and light-blue regions are UTRs. Intronic regions were analyzed for the presence of long interspersed repeats (shown in red), short interspersed repeats (shown in green), and long terminal repeats (shown in bright-pink).
