# Supplementary material for: Identification of the ancestral killer immunoglobulin-like receptor gene in primates
Source: BMC Genomics. 2006 Aug 15;7:209. doi: 10.1186/1471-2164-7-209 (PMC1559706; doi:10.1186/1471-2164-7-209)
Supplement: Additional File 2 — Sequence similarities between human KIR3DL1 and KIR3DL0 in primates. The table shows nucleotide and amino acid sequence similarities between human KIR3DL1 and KIR3DL0 in human, gorilla, chimpanzee, rhesus monkey and marmoset. [file 1471-2164-7-209-S2.pdf]

Additional file 2. Sequence similarities between human *KIR3DL1* and *KIR3DL0* in primates.

	% identity	
	**nucleotide**	**protein**
**human genomic**	61	40
**human cDNA**	50	33
**gorilla**	61	40
**chimpanzee**	61	39
**rhesus**	58	39
**marmoset**	62	41

For human *KIR3DL0*, both the cDNA and genomic sequence (manually corrected for the frameshift) have been used in the comparisons. The accession numbers for human *KIR3DL1* are AF022049 (nucleotide) and P43629 (protein). Multiple sequence alignments were carried out in EMBOSS (using Needle) with a gap opening penalty of 10 and a gap extension penalty of 0.5.
