# Supplementary figures and images for: Identification of the ancestral killer immunoglobulin-like receptor gene in primates
Source: BMC Genomics. 2006 Aug 15;7:209. doi: 10.1186/1471-2164-7-209 (PMC1559706; doi:10.1186/1471-2164-7-209)

Additional file 3. Domain-by-domain analyses of human KIR and LILR genes.

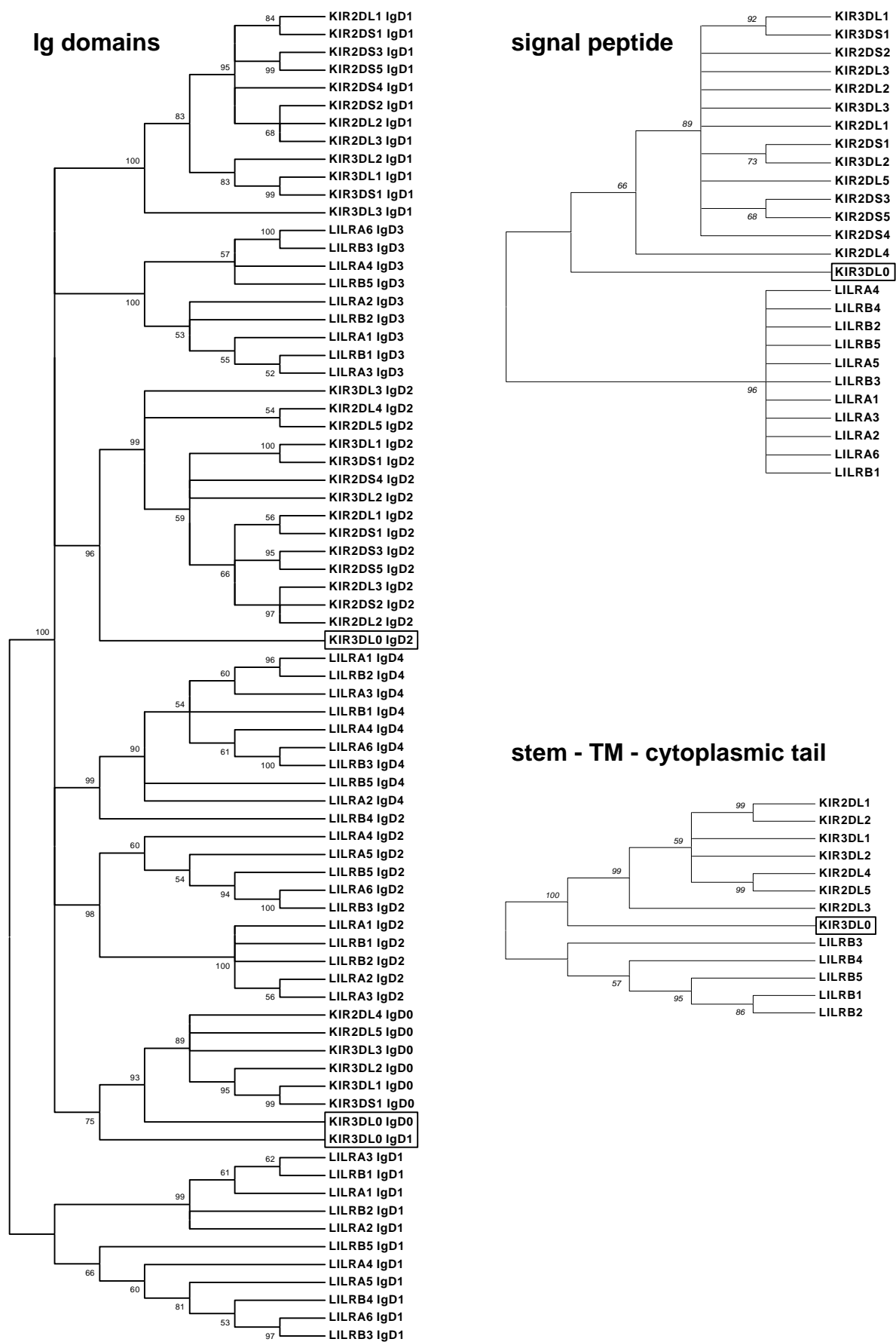

Supplement: Additional File 3 — Domain-by-domain analyses of human KIR and LILR genes. Phylogenetic trees are shown for the Ig domains, signal peptide, and the combined sequence of the stem, transmembrane and cytoplasmic tail. [file 1471-2164-7-209-S3.pdf]
